# Activation of CXCL16/CXCR6 axis aggravates cardiac ischemia/reperfusion injury by recruiting the IL‐17a‐producing CD1d^+^ T cells

**DOI:** 10.1002/ctm2.301

**Published:** 2021-01-27

**Authors:** Shijun Wang, Leilei Ma, Ji'e Yang, Zhen Dong, Jian Wu, Xiancheng Lu, Jiahong Wang, Fangjie Dai, Guowei Tu, Lei Xu, Gang Zhao, Feng Zhang, Yunzeng Zou, Junbo Ge

**Affiliations:** ^1^ Shanghai Institute of Cardiovascular Diseases, Zhongshan Hospital Fudan University Shanghai China; ^2^ Institutes of Biomedical Sciences Fudan University Shanghai China; ^3^ Department of outpatient Shanghai Yangpu Hospital of Traditional Chinese Medicine Shanghai China; ^4^ Department of Cardiology Yangpu Hospital, Tongji University School of Medicine Shanghai China

Dear Editor,

Reperfusion is commonly used for the treatment of acute myocardial infarction, but inflammation‐related reperfusion injury limits its clinical application. Blockade of IL‐17a is considered one of the effective therapies for myocardial ischemia/reperfusion (I/R) injury.^1^ However, the generation of cardiac IL‐17a and the subpopulations of IL‐17a‐producing cells remain obscure. In the present study, we reveal that the CXCL16/CXCR6 signal axis plays a key role in IL‐17a production and cardiac reperfusion inflammation in a mouse model of cardiac I/R injury.

CXCL16 is a proinflammatory chemokine that widely expressed in endothelial cells, vascular smooth muscle cells, and fibroblasts.^2^ CXCL16 is a novel diagnostic hallmark of acute coronary syndrome,^3^, ^4^ and CXCL16 is essential for the migration and recruitment of IL‐17a‐producing T cells to the atherosclerotic lesions.^5^ After myocardial ischemia for 40 min,  we observed that the protein level of CXCL16 increased rapidly in the left ventricle (LV) of mice at 2 h after reperfusion and maintained high level during the reperfusion period (Figure 1 A). With the highest abundance, CXCL16 in the heart stood out among those in the other organs after I/R (Figure 1 B), suggesting that myocardial I/R‐induced CXCL16 activation was cardiac specific. Moreover, we examined the transcriptional levels of a number of proinflammatory chemokines and their receptors in mouse heart with I/R stress. Compared with other chemokines, the mRNA levels of CXCL16, CCL5, CXCL1, and CXCR2 were significant upregulated after I/R (Figure S1A).To investigate the precise role of CXCL16 in cardiac I/R injury, we knocked down the endogenous CXCL16 by delivering adenovirus‐shRNA targeting CXCL16 gene 24 h before I/R injury (Figure S1B). The results showed that cardiac IL‐17a production (Figure 1 C) and cell apoptosis were significantly attenuated in CXCL16‐deficient mice compared with those in wild‐type (WT) mice (Figure 1 D and E). The severity of myocardial lesion is evaluated by two indexes, one is the ratio of the myocardial infarcted (INF) size to the size of area at risk (AAR) based on the method of Evans Blue and triphenyltetrazolium chloride (TTC) double staining, the other is the left ventricular ejection fraction (LVEF). Compared to mice transfected by scramble shRNA, INF/AAR reduced by 33% (30.83±6.72% vs. 46.04±15.18%, *p* < 0.05, Figure 1 F and G) and LVEF increased by 31% (44.79±4.33% vs. 58.43±5.37%, *p* < 0.05, Figure 1 H) in mice transfected by CXCL16 shRNA. Importantly, CXCL16 shRNA transfection in vivo also diminished the cardiac infiltration of CD1d^+^ monocytes in the heart under I/R (Figure 1 I). These data are consistent with recent published data in the hepatic I/R mouse model,^6^ indicating that suppression of CXCL16 ameliorated organ I/R injury through reducing the recruitment of inflammatory cells and cytokine production.

**FIGURE 1:**
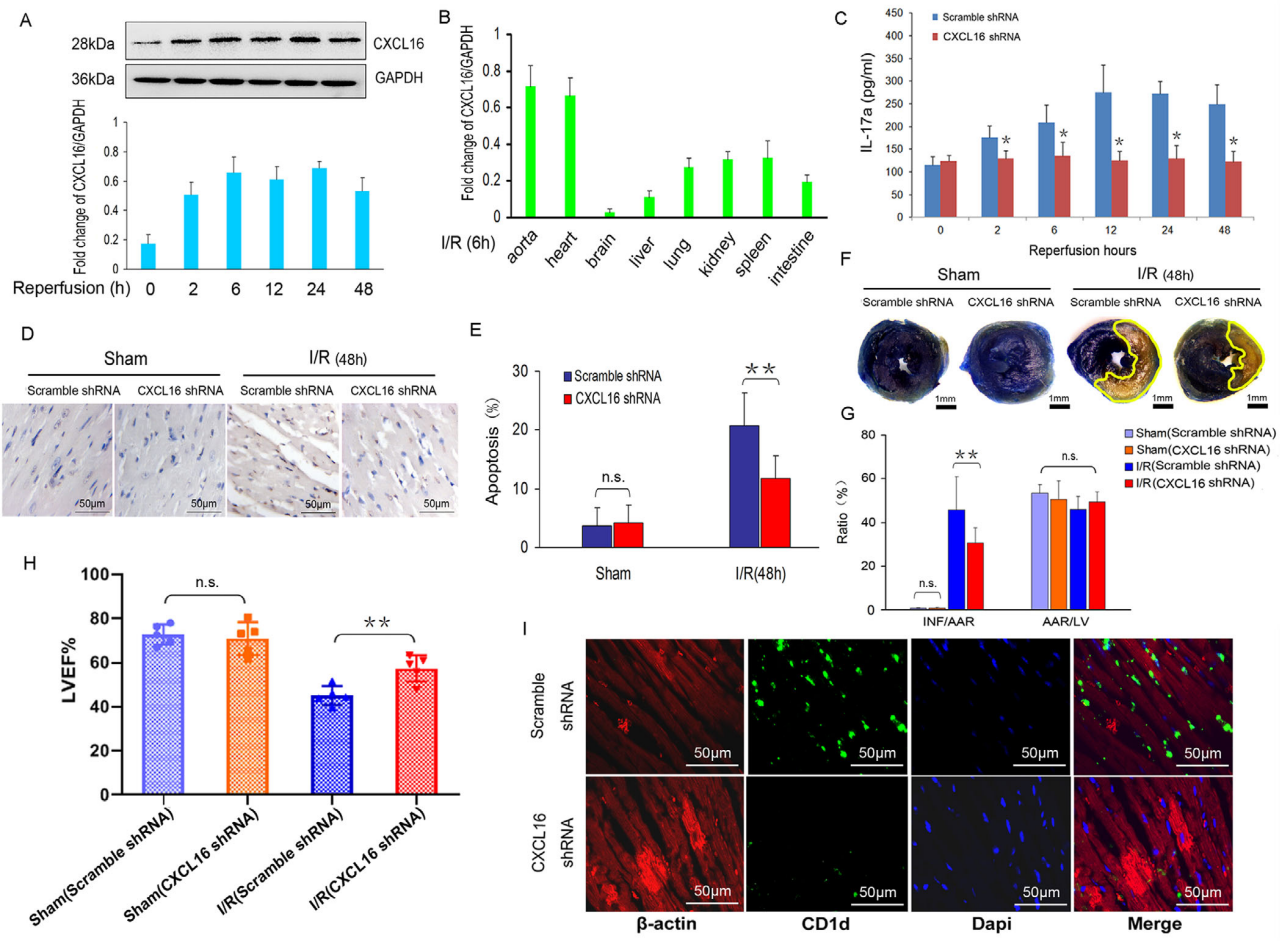
Inhibition of CXCL16 attenuated cardiac infiltration of CD1d^+^ cells, IL‐17a production, and I/R‐induced myocardial injury. Mice were subjected to 40‐min ischemia and followed by 48‐h reperfusion. (A) The protein levels of CXCL16 in the LV tissues of WT mice were examined at different time‐points after reperfusion. (B) Profiling of CXCL16 expressed on aorta, heart, brain, liver, lung, kidney, spleen, and intestine at 6 h after reperfusion. Then, mice were injected with recombinant adenoviruses delivered CXCL16 shRNA or scramble shRNA 24 h before I/R injury. (C) The serum level of IL‐17a was detected by ELISA. *p < 0.05 vs. scramble shRNA, *n* = 3 per group. (D) Cell apoptosis was detected by TUNEL staining. (E) Cell apoptosis was quantified by counting average number of the TUNEL positive cells in five randomly selected fields in border zone regions (*n* = 5, **p < 0.05, vs. I/R‐induced WT mice pretreated with scramble shRNA). (F) Representative images of heart cross sections stained by Evans blue and TTC double staining (Scale bar, 1 mm). (G) INF/AAR and AAR/LV were quantified (*n* = 4 per group, **p < 0.05). (H) Echocardiographic analysis of the cardiac function (LVEF) in different group of mice (*n* = 6, **p < 0.05). (I) Representative images showed that frozen sections of ischemic heart tissue stained by anti‐CD1d and anti‐β‐actin antibodies post I/R for 48 h

CXCR6 is the only specific chemokine receptor for CXCL16, previous studies imply that CXCR6 expression is essential for the activation, migration, and homing of CD1d‐restricted T‐cell subsets.^7^ Here, we aim to determine whether cardiac infiltrating CD1d^+^ monocytes are T‐subsets, whether these cells contribute to cardiac IL‐17a production, and whether disrupting the CXCL16/CXCR6 axis can prevent the recruitment of IL‐17a‐producing cells in the heart and attenuate myocardial I/R injury. By FACS sorting, we found a high proportion of IL‐17a‐producing CD3^+^ subpopulation in the heart tissue of I/R‐induced mice, while almost no CD3^−^ IL‐17a‐producing cells were found by isotype treatment (Figure 2 A), which further confirmed that T‐cell subpopulations were the major driving forces for IL‐17a production. Compared with WT mice, both CD45^+^ and CD3^+^ subpopulations were significantly reduced in CXCR6^−/–^ mice (Figure 2 B and C). Further, the proportions of IL‐17a‐producing T subsets, including IL23R^+^, CD1d^+^, and γδTCR^+^ cells, were all lower in CXCR6^−/–^ mice than in WT mice (Figure 2 D–F). Obviously, CXCR6 deficiency caused a pronounced reduction of CD1d^+^ IL‐17a‐producing T subset, which was confirmed by in situ immunofluorescence (Figure 2 G). These data suggested that blocking the CXCL16/CXCR6 axis was sufficient to abolish the recruitment of IL‐17a^+^ T cells, especially the CD1d^+^T subset, in the heart with I/R injury.

**FIGURE 2:**
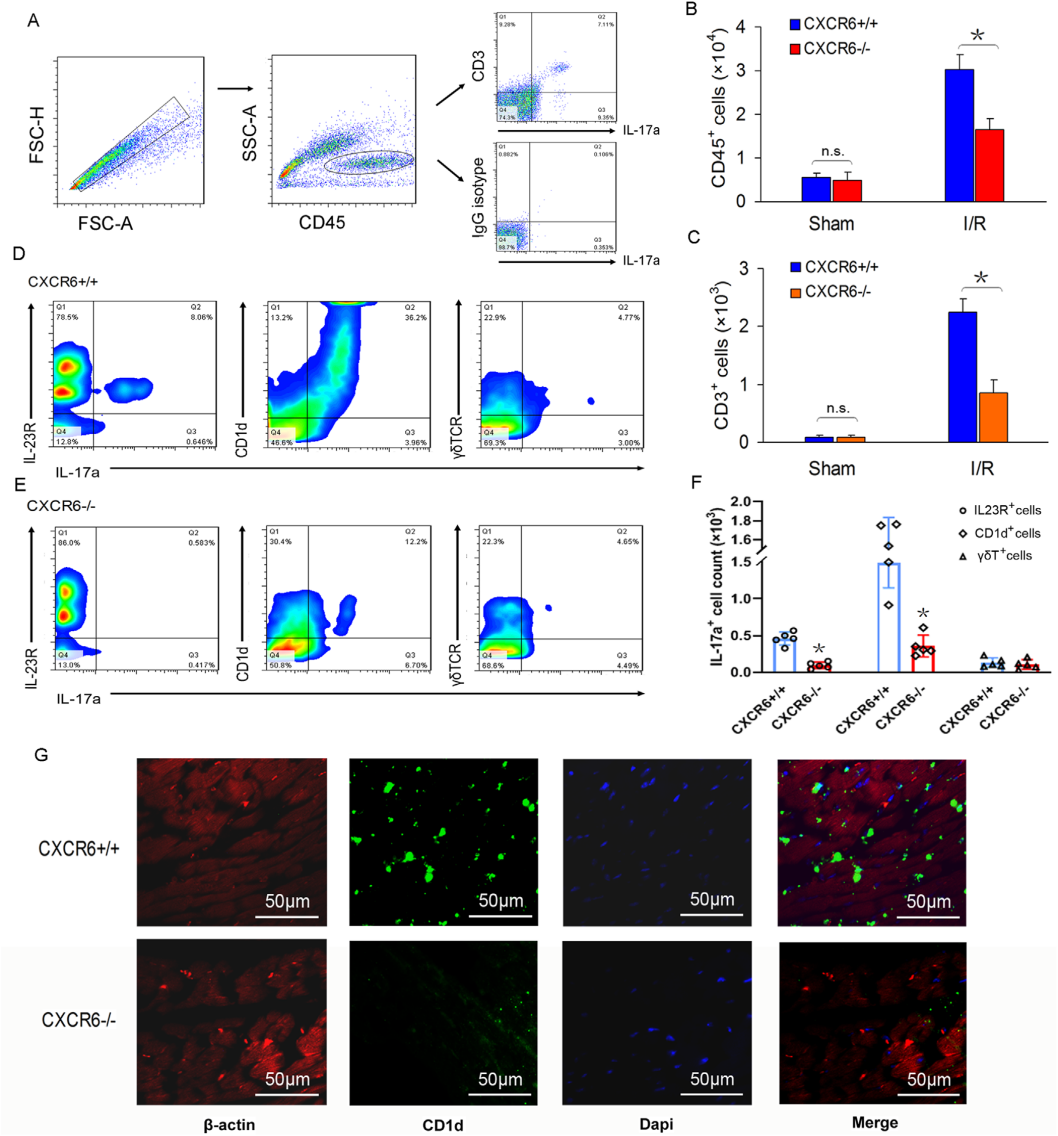
CXCR6 deficiency prevented cardiac infiltration of the IL‐17a‐producing T cells in response to I/R stress. Both CXCR6^−/−^ and wild‐type (WT) mice were subjected to 40‐min ischemia and followed by 48‐h reperfusion. The mice were then euthanized under (4%) isoflurane anesthesia and heart tissues were harvested. (A–C) The LV border zone tissue‐derived single‐cell suspension was isolated from CXCR6^−/−^ and WT mice, and cells were sorted by anti‐CD45 antibody, followed by gating on anti‐CD3 antibody or anti‐IgG1 isotype, and CD45^+^ subpopulation and CD3^+^ IL17a‐expressing subpopulation were quantified (*n* = 4, *p < 0.05 vs. WT mice with I/R). (D and E) Representative gate plots of cardiac IL‐23R^+^, CD1d^+^, and TCRγδ^+^ IL‐17‐producing T‐cell subsets in WT and CXCR6^−/−^ mice. (F) Quantification of IL‐17‐producing cells in different T‐subsets from WT and CXCR6^−/−^ mice (*n* = 4, *p < 0.05 vs. WT mice with I/R). (G) Immunofluorescence staining showed the representative frozen sections of ischemic heart tissue stained with both anti‐CD1d and anti‐β‐actin antibodies after cardiac I/R injury

Another finding was that circulating IL‐17a level increased in a time‐dependent manner in WT mice, while it maintained low level in CXCR6^−/–^ mice during reperfusion (Figure 3 A). Meanwhile, the expression of IL‐17a receptor (IL‐17RA) after I/R was significantly higher in WT mice than that in CXCR6^−/−^ mice (Figure 3 B). IL‐17a signaling is critically involved in I/R‐mediated organ injury, including heart, brain, lung, liver, kidney, and intestine.^1^, ^7^, ^8^ Secukinumab, a human‐derived monoclonal antibody against IL‐17a, has been approved in many countries for the therapy of ankylosing spondylitis, another IL‐17a neutralizing antibody (ixekizumab) and the antibody targeting IL‐17a receptor (brodalumab) have also been used in the treatment of inflammation‐related diseases.^9^, ^10^ Considering that cardiac infiltration of CD1d^+^ IL‐17a‐producing cells and IL‐17a production were significantly reduced in both CXCL16‐deficient mice and CXCR6^−/–^ mice, we further determined whether IL‐17a is critically involved in CXCL16/CXCR6 axis‐mediated cardiac I/R injury. The recombinant mouse IL‐17a (rIL‐17a) was injected into the heart of CXCR6^−/−^ mice at 6 h after reperfusion. After reperfusion for 48 h, cardiac cell apoptosis (Figure 3 C and D) and apoptotic‐related genes (Figure S1C and D) were significantly enhanced by rIL‐17a pretreatment. rIL‐17a also reduced LVEF by 26% (Figure 3 G) and increased INF/AAR by approximately 60% (48.94±14.37% vs. 30.68±5.46%, *p* < 0.05, Figure 3 E and F). In addition, we found that the survival benefit of CXCR6 deficiency was evident within 1 week post myocardial I/R surgery, but it was markedly reduced by the pretreatment with rIL‐17a (Figure 3 H). Collectively, current data suggest that IL‐17a acts downstream of CXCL16/CXCR6 signal axis, and IL‐17a plays a central role in I/R‐induced myocardial injury and dysfunction.

**FIGURE 3:**
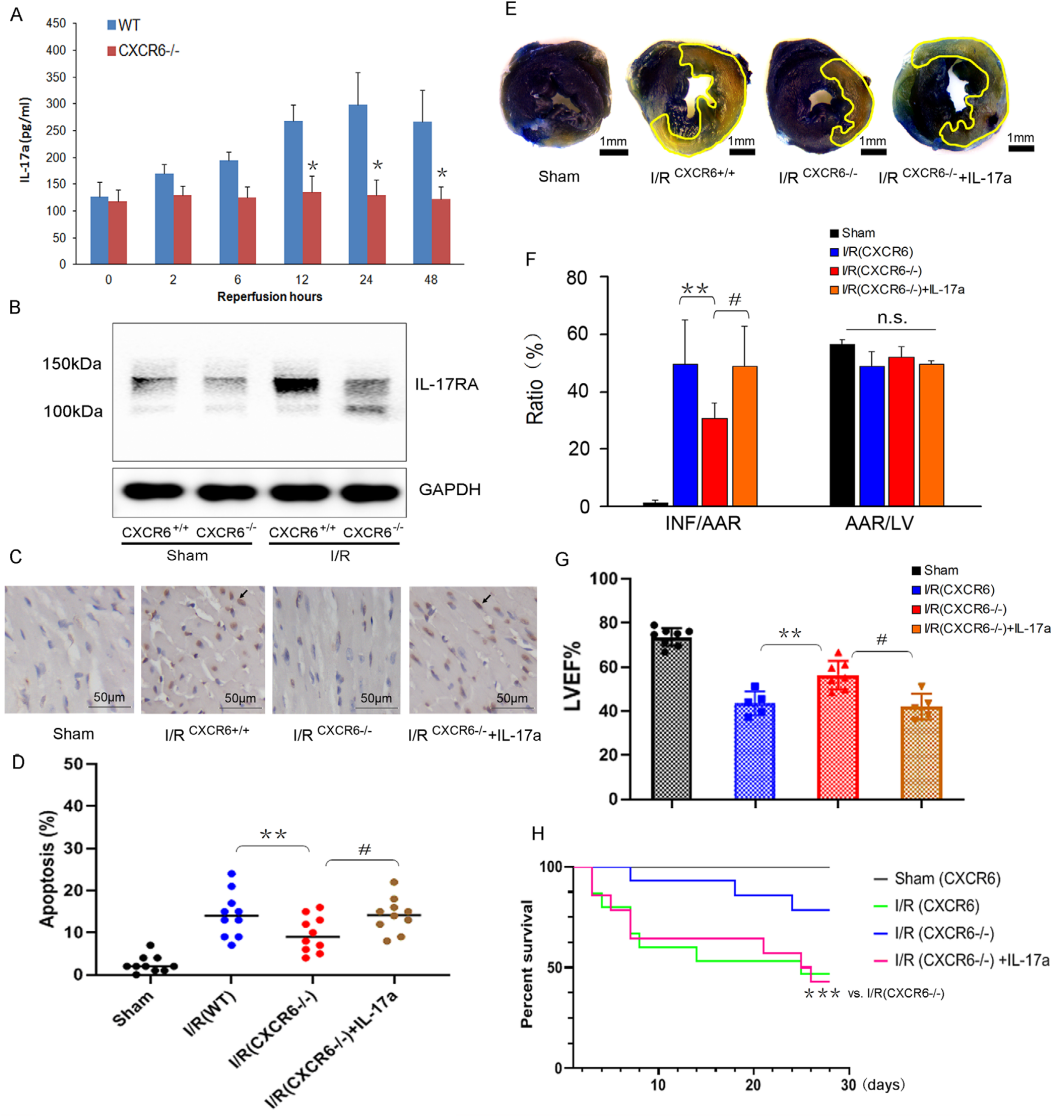
IL‐17a aggravated myocardial injury and dysfunction and increased the mortality of CXCR6^−/−^ mice in response to I/R stress. (A) The serum level of IL‐17a was determined at different time‐points after reperfusion, *n* = 3 per group, *p < 0.01 vs. WT mice. (B) The expression of IL‐17RA in the LV tissue of WT and CXCR6^−/−^ mice was determined by Western blot. Then, CXCR6^−/−^ mice were pretreated with recombined IL‐17a by ultrasound‐guided myocardial injection of rIL‐17a (10 ng/ml) in 20 μl 1 × PBS 2 h after reperfusion. (C and D) Representative images, infarct size relative to area at risk (INF/AAR, D, left), and AAR relative to left ventricle (AAR/LV, D, right) were shown by Evans blue and TTC double‐stained heart cross sections (Scale bar, 1 mm). *n* = 4 per group, **p < 0.05 vs. WT mice with I/R; ^#^ *p* < 0.05 vs. CXCR6^−/−^ mice with I/R. (E and F) Cardiac cell apoptosis was measured by TUNEL staining, and quantification of apoptotic cells by counting the number of TUNEL positive cells in 10 randomly selected fields from border zone regions (*n* = 10 per group, **p < 0.05, ^#^ *p* < 0.05). (G) Quantitative analysis of the left ventricular ejection fraction (LVEF) of mice by M‐mode echocardiograms (*n* = 5–8, ^**^ *p *< 0.05 vs. I/R‐induced WT mice, ^#^ *p *< 0.05 vs. I/R‐induced CXCR6^−/−^ mice, one‐way ANOVA with Tukey's post hoc test). (H) Kaplan–Meier analyses were performed to evaluate overall survival of sham‐operated or I/R‐mediated WT or CXCR6^−/−^ mice pretreated with or without IL‐17a (***p < 0.05 vs. CXCR6^−/−^ mice, log‐rank test)

In summary, our study demonstrates that I/R‐mediated CXCL16 activation promotes the recruitment of CXCR6^+^ IL‐17a‐producing T‐cell subsets in the heart, which enhances myocardial IL‐17a secretion and aggravates IL‐17a‐dependent cardiac remodeling and dysfunction. Considering the side effects of anti‐IL‐17a antibody in the treatment of autoimmune disorder, we think that CXCL16 might be a better candidate for targeted therapy of cardiac inflammation during reperfusion injury.

## CONFLICT OF INTEREST

The authors declare no conflict of interest.

## AUTHOR CONTRIBUTIONS

S.W. designed the study, performed most of the experiments, and wrote the paper. L.M., J.Y., and Z.D. performed the animal models. J.W. completed the echocardiographic evaluation of LV function. X.L and J.W. provided critical reagents. F.D., G.T., and L.X. analyzed and interpreted data. G.Z. and F.Z. provided critical reviews. Y.Z. and J.G. planned and supervised the project and data analysis of the experiments. All authors revised and approved the final manuscript.

## Supporting information

Supporting Information

Supporting Information

Supporting Information

Supporting Information

Supporting Information
