# Supplementary material for: Activation of CXCL16/CXCR6 axis aggravates cardiac ischemia/reperfusion injury by recruiting the IL‐17a‐producing CD1d+ T cells
Source: Clin Transl Med. 2021 Jan 27;11(2):e301. doi: 10.1002/ctm2.301 (PMC7839957; doi:10.1002/ctm2.301)
Supplement: Supplementary file 1 — Supporting Information [file CTM2-11-e301-s001.docx]

**Supplemental Figure legends**

**FIGURE S1** A, Quantification of the mRNA levels of chemokines and chemokine receptors before and after cardiac I/R injury. Data are presented as mean±s.e.m. n = 3, *P < 0.05 vs. sham groups, analyzed by Two-way ANOVA followed with multiple comparisons test. I/R induced the transcriptional upregulation of CCL5, CXCL1, CXCL4, CXCL12, CXCL16 and CXCR2, and CCL5, CXCL1, CXCL16 and CXCR2 showed the significant difference between sham group and I/R group, while the transcriptions of CCL2, CCL7, CCR2, CCR6 and CXCR4 were not changed by cardiac I/R. B, Western blot showed the protein expression of CXCL16 in the LV tissue of mice pretreated with CXCL16 shRNA or scramble shRNA. C-D, Quantitative analysis of the transcriptional levels of Bcl-2 and p53 in sham-operated WT mice, I/R-induced WT mice, I/R-induced CXCR6^-/-^ mice and I/R-induced CXCR6^-/-^ mice with IL-17a pretreatment (n=10, **P*<0.05 vs. sham-operated WT mice, ***P*<0.05 vs. I/R-induced CXCR6^-/-^ mice, ^#^*P*<0.05 vs. I/R-induced CXCR6^-/-^ mice).
