# Supplementary figures and images for: Activation of CXCL16/CXCR6 axis aggravates cardiac ischemia/reperfusion injury by recruiting the IL‐17a‐producing CD1d+ T cells
Source: Clin Transl Med. 2021 Jan 27;11(2):e301. doi: 10.1002/ctm2.301 (PMC7839957; doi:10.1002/ctm2.301)

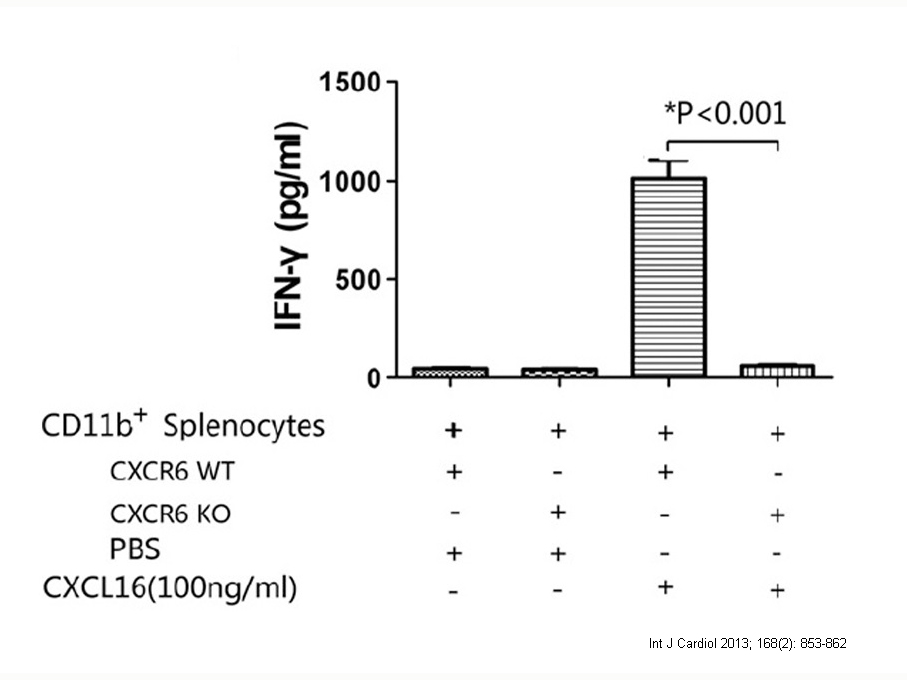

Supplement: Supplementary file 2 — Supporting Information [file CTM2-11-e301-s002.jpg]

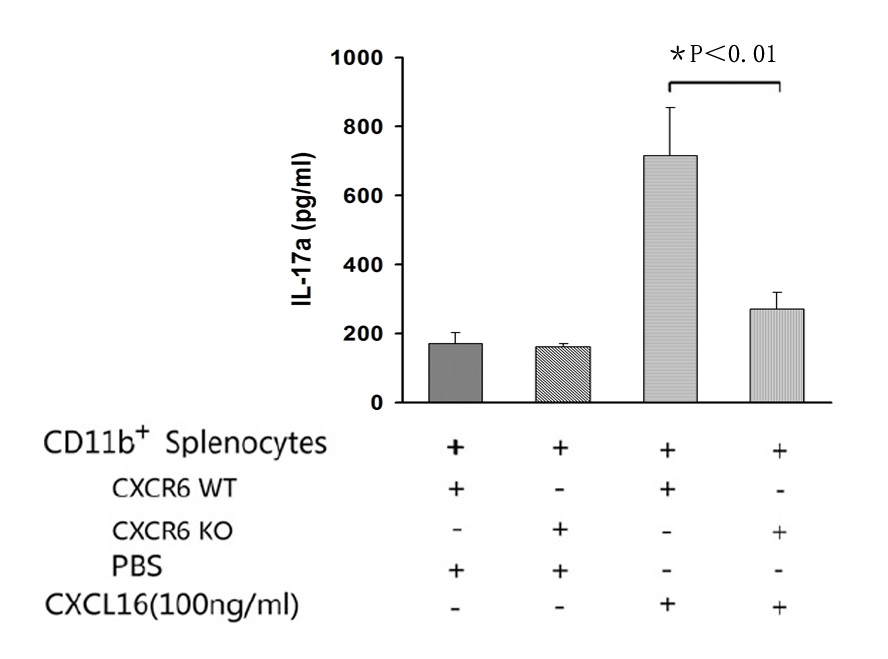

Supplement: Supplementary file 3 — Supporting Information [file CTM2-11-e301-s003.jpg]

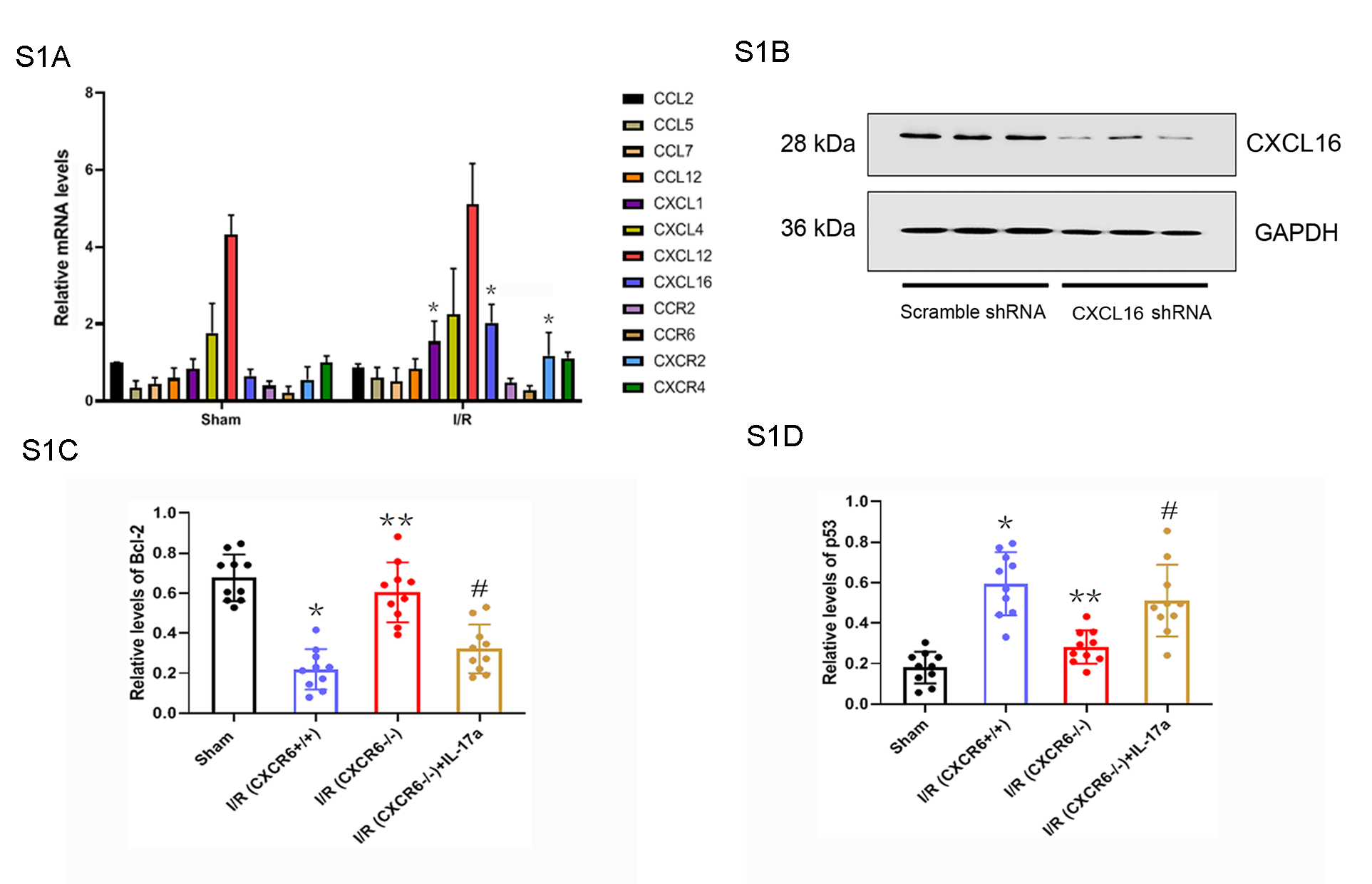

Supplement: Supplementary file 4 — Supporting Information [file CTM2-11-e301-s004.tif]
