# Supplementary material for: Activation of CXCL16/CXCR6 axis aggravates cardiac ischemia/reperfusion injury by recruiting the IL‐17a‐producing CD1d+ T cells
Source: Clin Transl Med. 2021 Jan 27;11(2):e301. doi: 10.1002/ctm2.301 (PMC7839957; doi:10.1002/ctm2.301)
Supplement: Supplementary file 5 — Supporting Information [file CTM2-11-e301-s005.doc]

**Supplemental Table S1 List of primers used for mRNA QT-PCR.**

| **Gene ID** | **Forward (5’-3’)** | **Reverse (5’-3’)** |
| --- | --- | --- |
| CCL2 (20296) | agcaccagccaactctcact | tcattgggatcatcttgctg |
| CCL5 (20304) | ccctcaccatcatcctcact | gagcacttgctgctggtgta |
| CCL7 (20306) | tgaaaaccccaactccaaag | ttaggcgtgaccatttcaca |
| CCL12 (20293) | tcctcaggtattggctggac | gggtcagcacagatctcctt |
| CXCL1 (14825) | gctgggattcacctcaagaa | cttggggacaccttttagca |
| CXCL4 (56744) | agccctagacccatttcctc | gatctccatcgctttcttcg |
| CXCL12 (20315) | gctctgcatcagtgacggta | taatttcgggtcaatgcaca |
| CXCL16 (66102) | agcgcaaagagtgtggaact | ggttgggtgtgctctttgtt |
| CCR2 (12772) | tttgcaactgcctctttcct | cttctgtccctgcttcatcc |
| CCR6 (12458) | ggctctcccatccacataga | tggcacaaataccttggtga |
| CXCR2 (12765) | ggtggggagttcgtgtagaa | cgaggtgctaggatttgagc |
| CXCR4 (12767) | aaagctagccgtgatcctca | caccatttcaggctttggtt |
| p53 (22059) | tgctcaccctggctaaagtt | actcctccatggcagtcatc |
| Bcl-2 (12043) | agcattgcggaggaagtaga | ttcttggatgaaggggtgtc |
